# Authenticity of Hay Milk vs. Milk from Maize or Grass Silage by Lipid Analysis

**DOI:** 10.3390/foods10122926

**Published:** 2021-11-26

**Authors:** Sebastian Imperiale, Elke Kaneppele, Ksenia Morozova, Federico Fava, Demian Martini-Lösch, Peter Robatscher, Giovanni Peratoner, Elena Venir, Daniela Eisenstecken, Matteo Scampicchio

**Affiliations:** 1 Faculty of Science and Technology, Free University of Bozen-Bolzano, Piazza Università 5, 39100 Bolzano, Italy; Sebastian.imperiale@natec.unibz.it (S.I.); ksenia.morozova@unibz.it (K.M.); 2 Laimburg Research Centre, Laimburg 6, Pfatten-Vadena, 39040 Auer-Ora, Italy; elke.kaneppele@student.uibk.ac.at (E.K.); federico.fava@laimburg.it (F.F.); demian.martini-loesch@laimburg.it (D.M.-L.); peter.robatscher@laimburg.it (P.R.); giovanni.peratoner@laimburg.it (G.P.); elena.venir@laimburg.it (E.V.); daniela.eisenstecken@laimburg.it (D.E.)

**Keywords:** bovine feeding, LC-MS, milk, lipidomics, silage, hay milk, GC-MS, food authenticity, cyclopropane fatty acids, CPFAs

## Abstract

Hay milk is a traditional dairy product recently launched on the market. It is protected as “traditional specialty guaranteed” (TSG) and subjected to strict regulations. One of the most important restrictions is that the cow’s feed ration must be free from silage. There is the need for analytical methods that can discriminate milk obtained from a feeding regime including silage. This study proposes two analytical approaches to assess the authenticity of hay milk. Hay milk and milk from cows fed either with maize or grass silage were analyzed by targeted GC-MS for cyclopropane fatty acid (dihydrosterculic acid, DHSA) detection, since this fatty acid is strictly related to the bacterial strains found in silage, and by HPLC-HRMS. The presence of DHSA was correlated to the presence of maize silage in the feed, whereas it was ambiguous with grass silage. HPLC-HRMS analysis resulted in the identification of 14 triacylglycerol biomarkers in milk. With the use of these biomarkers and multivariate statistical analysis, we were able to predict the use of maize and grass silage in the cow’s diet with 100% recognition. Our findings suggest that the use of analytical approaches based on HRMS is a viable authentication method for hay milk.

## 1. Introduction

In recent years, there has been an increasing interest to sustain and develop European mountain areas and decrease land abandonment [1]. Mountain dairy farming is more challenging due to harsher environmental and morphological conditions, which lead to higher workload and management costs [2]. To counteract the economical disadvantages derived from natural constraints in these areas, the European Union (EU) is applying new policies to promote the quality and authenticity of mountain products [1,3]. The EU scheme “traditional specialty guaranteed” (TSG) represents an important policy for the valorization of traditional products. 

Hay milk is one of the dairy products that received the TSG label and is subjected to strict production regulations. This product is obtained with traditional methods [4], and is perceived by consumers as healthier and more natural [5]. Hay milk has been regaining popularity in recent years, especially in the alpine region [2,4,5], thanks to marketing, labelling, and certification strategies. This effort was made to valorize and differentiate local mountain production and to fully benefit from the TSG label [2]. 

In the specific case of hay milk, its TSG designation is controlled by the European Commission regulation 2016/304 [6]. It states that any form of fermented fodder, i.e., silage from maize and grass, moist hay, fermented hay, and any genetically modified feed is banned. Roughage, including fresh herbage and hay, but possibly also green rapeseed, green maize, green rye, and fodder beets, as well as hay, lucerne, maize pellets and similar types of feed, must make up at least 75% of the yearly ration of dry feed [6].

Even though the above regulation on the feeding is very clear, to the best of our knowledge, no clear markers or analytical methods for milk analysis are available to determine the presence of fermented fodder in the feed ration, especially for grass silage.

Some efforts to discriminate milk authenticity have been reported recently, especially through the analysis of the milk fat fraction, which is likely the most affected by the animal’s diet [7]. For instance, it has been shown that different diets influence the fatty acids profile of milk fat [8]. Additionally, fatty acid characterization can provide information related to both diet composition and the ruminal fermentation pattern. Finally, many studies aimed to identify markers within this lipid class [9,10,11,12,13,14]. 

Recent literature reported the analysis of a group of specific fatty acids (cyclopropane fatty acids, CPFAs) used as markers for the authenticity of Parmigiano Reggiano cheese [9]. Indeed, similarly to hay milk, Parmigiano Reggiano cheese is produced with milk from cows fed without any silage [15]. In that work, CPFAs were found only in dairy products obtained with maize silage in the cow’s diet [9]. Later, a method was proposed and validated [11], and CPFAs have been included in the Parmigiano Reggiano PDO regulations for the verification of its authenticity. Lactic acid bacteria (LAB) produce CPFAs as response to fermentative stress [16,17,18,19,20]. LAB convert soluble carbohydrates, present in matrices rich in starch like maize, into lactic acid [21]. However, it is not clear if the proposed method is also suitable to determine the presence of grass silage in the animal’s diet.

For this reason, this study investigates the capacity of the CPFA method to assess the authenticity of hay milk with respect to milk obtained from cows fed with maize and, to the best of our knowledge, for the first time, with grass silage in the feed ration. Furthermore, this work proposes the use of an approach based on high-performance liquid chromatography (HPLC), for the detection and identification of new markers, or groups of markers. HPLC has advantages such as high analysis speed, resolution, and sensitivity [22]. When coupled to high-resolution mass spectrometry (HRMS), HPLC represents a powerful tool to analyze the composition of milk fat, especially for untargeted studies. Several lipidomic studies use HPLC and mass spectrometry to characterize milk lipids from cows fed with different diets [23,24]. Craige Trennery et al. performed LC-MS to study the effects of feeding on the milk lipid profile [25]. Because of the high amount of data obtained by HPLC-HRMS, the implementation of chemometrics and multivariate statistical methods is essential to elucidate the characteristics of milk.

For these purposes, this work analyzed three types of milk, namely hay milk (HM), milk from cows fed with maize silage in the ration (SM-M), and milk from cows fed with grass silage in the ration (SM-G), by GC-MS and HPLC-HRMS. The first part of the study followed a targeted approach to determine the amount of the CPFA dihydrosterculic acid (DHSA) in each milk sample, using a GC-MS method adapted from Marseglia et al. [9]. In the second part, HPLC-HRMS was used to characterize the lipid profile of each sample. Untargeted pattern recognition and correlation of the different feeding practices were conducted using multivariate statistical analysis. The combination of the presence of DHSA with multivariate analysis on the resulting HRMS data provided an analytical fingerprint that allows discrimination among the forages implemented in dairy farming.

## 2. Materials and Methods

### 2.1. Milk Samples 

**Figure 1:**
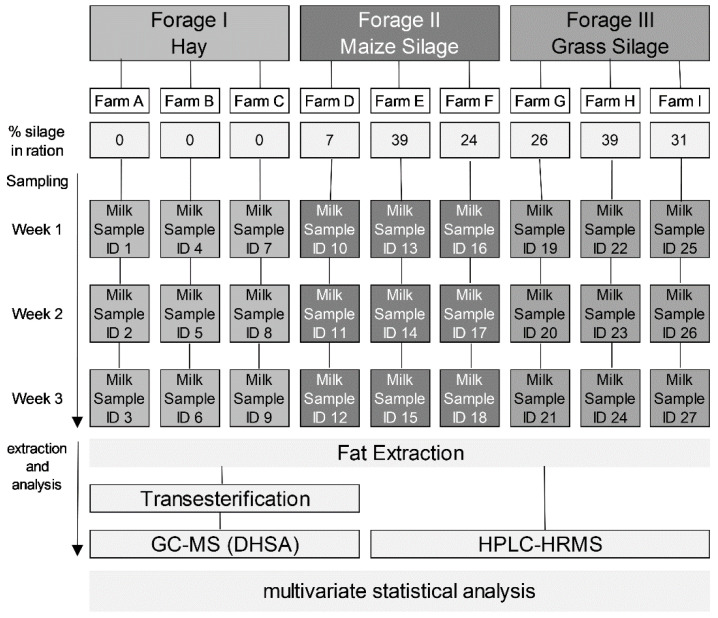
Sampling scheme for targeted GC-MS and HPLC-HR MS analysis of milk. Nine farms were selected based on the feeding regimen. Each sample is shown in the figure.

A total of 27 fresh and unpasteurized bulk milk samples were collected from 9 dairy farms located in the north of Italy (South Tirol, Italy), at altitudes ranging from 616 m a.s.l. to 1404 m a.s.l. Sample collection was dispersed over the winter feeding interval from October 2019 to March 2020. At each farm, bulk milk was sampled weekly over three consecutive weeks, in order to capture variability over time (Figure 1). 

The farmers were interviewed by means of a structured survey concerning several aspects of the farm structure, characteristics, and the composition of the feed ration during the sampling period, and the proportion of silage in the diet was computed on a dry matter basis. At three farms, the animals were fed according to the EU-Regulation 2016/304 of hay milk production, i.e., without fermented fodder and roughage making up at least 75% dry matter of the yearly ration (hay milk, HM). The other six farms included silage in amounts ranging from 7% to 39% dry matter of the total feed ration: three included only maize silage (maize silage milk, SM-M), and three only grass silage (grass silage milk, SM-G). Each farm had between 16–35 cows. The milk samples were taken in the morning from the farm’s own milk tank, being therefore a mix of the evening and morning milk. Before taking the samples, the milk was mixed by hand and then the samples were taken with a liquid sampler. At each sampling event, aliquots of 30 mL for each sample were taken from the same tank for the analysis of DHSA, for the lipid profile via HPLC-HRMS and for quality routine analysis, respectively. During transportation, the samples were kept refrigerated at a temperature of about 4 °C before being stored at −80 °C until analysis.

### 2.2. Chemicals and Reagents

Methanol was purchased from VWR (Radnor, PA, USA), and pentane was obtained from Fluka Analytical (Honeywell International Inc., Charlotte, NC, USA). Hexane and sodium methoxide solution 25 wt.% were purchased from Sigma Aldrich (St. Louise, MO, USA). Sodium sulfate was purchased from Titolchimica (Rovigo, Italy, IT) and the CPFA cis-9,10-methylene-octadecanoic acid (dihydrosterculic acid DHSA, as methyl ester, purity ≥98%) was obtained from Chem Cruz (TE Huissen, The Netherlands, NL). LC-MS grade formic acid and LC-MS grade ammonium formate were purchased from Sigma Aldrich (Steinheim, Germany). LC-MS grade methanol and LC-MS grade acetonitrile were purchased from Honeywell (Selze, Germany), and LC-MS grade 2-propanol and methyl tert-butyl ether (MTBE) were purchased from Merck KGaA (Darmstadt, Germany) and not purified further. If not otherwise stated, Milli-Q water was employed.

### 2.3. Sample Preparation and Analysis via GC-MS

#### 2.3.1. Milk Fat Extraction 

The milk samples were thawed in a water bath at 40 °C for 2 h. The fat was separated following a modified method based on Feng et al. [26]. A volume of 20 mL of milk was added in a 50 mL conical plastic tube and centrifuged at 12,000 rpm (17,800× *g*) for 30 min at 4 °C. After centrifugation, the fat cake (top layer) was transferred into a 15 mL conical plastic tube and stored over night at −80 °C. The fat was resuspended in a volume of 10 mL of a 9:1 (*v*/*v*) n-pentane/methanol, then vortexed for 2 min at room temperature and kept in a room temperature ultrasound bath (45 kHz) for 5 min, shaken for 5 min with a MultiRotator (PTR-60 Grant Intruments, Royston, UK), then vortexed again for 2 min with a final centrifugation at 4000 rpm (1900× *g*) for 2 min at room temperature. The organic phase was transferred into a dark glass vial and flushed with N_2_ until dryness. The fat was stored at −80 °C until transesterification.

#### 2.3.2. Transesterification

Transesterification was carried out according to Christie et al. [27]. Milk fat (100 mg ± 5 mg) was dissolved in 5 mL hexane. Then, 0.2 mL of sodium methoxide in dry methanol (1 mL sodium methoxide solution 25 wt% diluted with 1.25 mL methanol) was added, and the solution was briefly agitated (30 s) to ensure thorough mixing. The reaction was quenched by adding 0.5 g sodium sulfate, and after a brief agitation (30 s), it was centrifuged at 2000× *g* for 5 min at room temperature and the supernatant was used for analyses.

#### 2.3.3. Analysis of the Cyclopropane Fatty Acid Dihydrosterculic Acid (DHSA) 

The GC-MS analysis was carried out on a Shimadzu GC MS-QP2010 SE (Kyoto, Japan) equipped with an autosampler, a split/splitless injection port, a GC oven and a single quadrupole mass spectrometer. Each sample was measured in triplicate. For the analyses, 100 µL of transesterified mixture was taken, diluted with 900 µL hexane, and 1 µL was injected using a split ratio of 1:10. Helium was used as carrier gas with a flow rate of 1 mL/min and a low-polarity SLB-5 ms column (30 m × 0.25 mm i.d × 0.25 µm) (Supelco, Bellefonte, PA, USA) was used for chromatographic separation of the analyte. The run was conducted following a modified temperature program according to Marseglia et al. [9]. Temperature was kept at 40 °C for 5 min, increased at 280 °C with a rate of 10 °C/min and held for 10 min. The injector temperature and transfer line temperature were maintained at 280 °C and ion source temperature at 230 °C. The mass spectra were acquired in full scan mode (mass range 40–500 *m*/*z*) and in SIM Mode (using 55 *m*/*z* as quantifier, 69, 278, and 310 *m*/*z* qualifier). The quantification of DHSA in the samples was carried out by comparing the peak area of the samples with the peak area of known amounts of the DHSA standard, considering the matrix effect by spiked hay milk with DHSA following extraction and transesterification. The limit of detection (LOD) of the method was 7.5 mg DHSA/kg of fat and the limit of quantification (LOQ) was 25.0 mg DHSA/kg of fat. Linear range was from 25.0 mg/kg to 1500 mg/kg. Recovery of spiked fat was 101.5% (0.2 RSD%). Intraday repeatability was of 3.3, 5.4, 2.5 RSD% for 80, 400, and 1000 mg DHSA/kg of milk fat, respectively.

### 2.4. Sample Preparation and Analysis via HPLC-HRMS

#### 2.4.1. Milk Fat Extraction

The milk samples were thawed at 8 °C overnight. Fat extraction from milk samples was carried out according to Breitkopf et al. based on the extraction method by Matyash et al. with modifications [28,29]. In short, 200 μL of milk was pipetted into a 15 mL centrifuge tube, 1.5 mL methanol was added and vortexed for 1 min. Then, 5 mL of MTBE was added and shaken at 200 rpm for 1 h at room temperature. After the incubation, 1.2 mL of water was added and vortexed for 1 min. The mixture was centrifuged for 10 min at 1000× *g* at room temperature. The upper phase was collected, and the bottom phase re-extracted with 2 volume parts of MTBE/methanol/water (10/3/2.5, *v*/*v*/*v*). The combined upper phases were dried under nitrogen flow at room temperature (MultiVap 8, LabTech S.r.l., Milano, Italy). Finally, the dried extract was dissolved in 5 mL methanol/2-propanol (50/50, *v*/*v*), diluted 1:100 with the same solvent mix and filtered with a 0.45 μm PTFE syringe filter prior to injection.

#### 2.4.2. High-Performance Liquid Chromatography Coupled to High Resolution Mass Spectrometry (HPLC-HRMS)

The system consisted of a Q-Exactive hybrid quadrupole Orbitrap HRMS instrument (Thermo Fisher Scientific, Waltham, MA, USA) coupled to an Ultimate 3000 UHPLC instrument (Thermo Fisher Scientific, Waltham, MA, USA) with UV-vis detector. The separation of the compounds was done at a flow rate of 0.2 mL/min with a C18 column (Accucore RP-MS, 100 mm × 2.1 mm i.d., 2.6 μm particle size, Thermo Fisher Scientific, Waltham, MA, USA) with a security guard cartridge system (Thermo Fisher Scientific, Waltham, MA, USA). The mobile phase consisted of a combination of solvent A (water/acetonitrile 40/60 *v*/*v* with the addition of 0.1% formic acid and 10 mM ammonium formate) and B (acetonitrile/2-propanol 10/90 *v*/*v* with addition of 0.1% formic acid and 10 mM ammonium formate). The gradient was set as follows: 70 % B (*v*/*v*) for 2 min, then from 70 % B to 83 % B at 3 min, hold until 8 min then to 84 % B at 13 min and hold until 14 min. Sample injection volume was 5 μL using an autosampler with a 20 μL injection loop. After each sample, a wash step with a blank (2-propanol) was introduced with the same chromatographic set-up as before, but with a different gradient: from 84 % B at 0 min to 97 % B at 2 min, hold 97 % until 7 min, from 97% at 7 min to 70 % B at 8 min followed by a re-equilibration step (70% B) from 8 to 10 min. Blank injection volume was 20 μL. During this wash and re-equilibration step, the flow from the HPLC was diverted to waste using a Rheodyne switch valve, while a flow of 3 μL/min 2-propanol was delivered to the MS using an infusion syringe pump (Thermo Fisher Scientific, Waltham, MA, USA) to avoid clogging and minimize carry-over effects. The HRMS instrument was operated in positive ionization mode with a heated electrospray ionization ion source set as follows: sheath gas flow at 40 (arbitrary units), aux gas flow at 10 (arbitrary units), sweep gas flow at 0 (arbitrary units), spray voltage at 4.00 kV, capillary temperature at 300 °C, S-lens RF level at 50%, and aux gas temperature at 100 °C. Full-MS experiments were performed in a scan range from 150 to 1500 *m*/*z* with a resolution of 35,000 (at 200 *m*/*z*), an automatic gain control (AGC) target of 2 × 10^5^ and a maximum injection time (IT) of 200 ms. Targeted SIM (t-SIM) experiments were performed with a resolution of 35,000, AGC target of 2 × 10^5^, max IT of 125 ms, and an isolation window of 4 *m*/*z*. The MS^2^ measurements of the selected ions were performed with a resolution of 17,500 and AGC target set at 1 × 10^5^ and maximum IT of 50 ms, with a stepped normalized collision energy of 20, 30, and 60 eV.

### 2.5. Data Processing and Statistical Analysis

Correlation of chemical compounds relative abundances and integration of the area under each peak (HPLC-MS XIC integrations) was done using Compound Discoverer 3.1 and Xcalibur (Thermo Scientific, Milano, Italy) and by employing online (LIPIDMAPS) and local databases. 

Multivariate statistical analysis was conducted using XLSTAT annual version 2021.1.1 1092 (Addinsoft 2021, New York, NY, USA).

## 3. Results and Discussion

### 3.1. GC-MS Analysis of DHSA

Table 1 shows the results of the determination of the CPFA dihydrosterculic acid (DHSA) in milk samples grouped into three categories: HM (hay milk, i.e., cows fed without the use of silage), SM-G (milk obtained from cows fed with grass silage in the ration), and SM-M (milk obtained from cows fed with maize silage in the ration).

As expected, and reported in a previous work [9], no CPFA, in this case DHSA, was detected in the HM samples, whereas it was detected in all SM-M samples. According to Caligiani et al., their GC-MS method was proposed as analytical tool for the detection of the marker in milk for the presence of silage in the feed ration of the cows [11]. The results obtained in the current study highlights how, for the SM-G samples, the determination of the DHSA is ambiguous. Indeed, in this study DHSA was not detected in four of the nine SM-G samples (three in farm H, and one in farm G). 

These limitations might be due to a series of factors, one of them being the sensitivity of the method in detecting the marker. For the method employed for DHSA detection in milk fat, a limit of detection (LOD) of 7.5 mg/kg and a limit of quantification (LOQ) of 25.0 mg/kg were obtained. The concentrations of DHSA found in the SM-G samples were generally lower than in the SM-M samples. Supposedly, a lower amount of available carbohydrates, such as during grass fermentation might lead to a reduced LAB stress response and to lower or no content of DHSA in the milk samples produced from grass silage in the ration. Overall, the results obtained with GC-MS allow us to discriminate HM vs. SM-M samples, but they were unable to discriminate all SM-G samples from the HM samples. For this reason, an approach using HPLC-HRMS was further proposed for the discrimination of milk obtained with different types of forages during the winter feeding period for the 27 samples collected.

### 3.2. Milk Fat Analysis by HPLC-HRMS 

**Figure 2:**
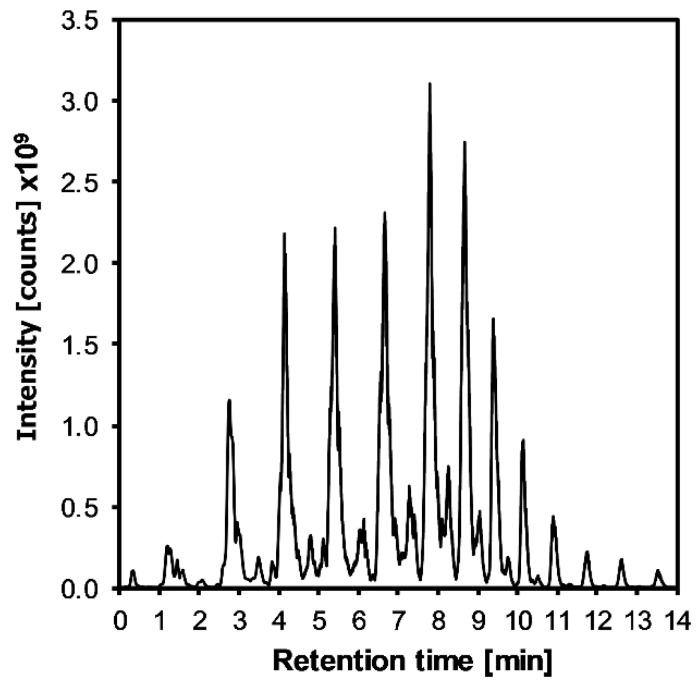
Total ion chromatogram acquired in full-MS showing the lipid profile of a milk fat extract obtained by HPLC-HRMS in positive ionization mode.

For non-target milk fat analysis, the milk fat profiles were determined using HPLC-HRMS in full-scan mode (full-MS, 150 to 1500 *m*/*z*). Figure 2 shows a typical total ion current (TIC) chromatogram obtained from a milk fat sample in positive ionization mode. For every sample of each milk type, the lipid profile was obtained.

For each profile, distinct cluster peaks could be observed representing a unique fingerprint of each milk sample. With HRMS it is possible to obtain the whole lipidome of milk [28]. However, in order to find the target markers that could be used to distinguish milk samples with different feeding, the use of chemometric tools was needed to process the obtained data. This was achieved by using a software for untargeted MS analysis (Compound Discoverer 3.1). With Compound Discoverer it was possible to build up a peak table with the most abundant masses for all analyzed samples and match the compounds with online and custom databases. 

**Figure 3:**
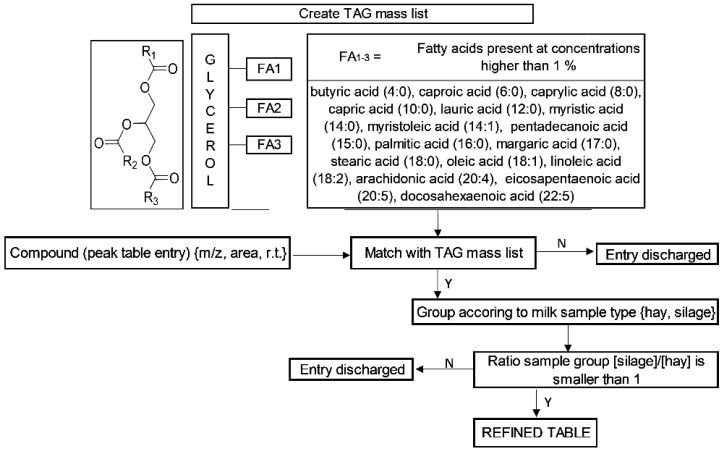
Creation of triacylglycerol (TAG) mass list and implemented algorithm for the identification of target molecules using HRMS. FA = Fatty Acid, N = no, Y = yes, r.t. = retention time.

The custom database in the software included a mass list with possible compounds of interest. In detail, we created a mass list comprising the most abundant lipid class in milk, the triacylglycerols (TAGs) which account for hundreds of different species (Figure 3) [30]. TAGs are composed of a glycerol molecule esterified with three fatty acids, which can be the same or different. When using the 16 fatty acids (FA) most common in milk [31], randomly distributed, it was possible to calculate 4096 (16^3^) theoretical TAGs and their corresponding *m*/*z* values. Considering isomers with identical exact mass, the list was reduced to 253 groups of TAG molecular species, which contained the same number of carbons (CN) and the same number of double bonds (DB) in their FA residues (Supplementary Table S1). 

The custom database with 253 masses of selected groups of TAG molecular species was used to match the masses in the peak table with the highest abundancy detected in the milk fat samples (Figure 3). All compounds that did not match the mass list were discarded. For the further data analysis, only these groups of TAG molecular species were selected (232 masses).

The scope of the study was to discriminate between hay milk and non-hay milk samples. For this reason, the relative intensities of matched groups of TAG molecular species were grouped in hay and silage samples. The extracted ion chromatograms of the selected TAG molecular species were generated with the following integration of the peaks. Variation in the TAG profiles between sample groups were reflected in the relative areas. Increase or decrease of matched TAG molecular species in one group could be used to differentiate between hay and silage samples. Therefore, we calculated the ratio of each area between the silage group and the hay group. All TAG molecular species that demonstrated a ratio inferior to one between groups were selected as potential markers to create the refined peak table (Figure 3). This allowed us to identify 14 groups of TAG molecular species that demonstrated the biggest differences between hay vs. silage sample groups (Table 2).

### 3.3. Tentative Identification of TAGs Marker 

Tentative identification of the 14 groups of TAG molecular species was performed using a data-dependent HPLC-HRMS-MS^2^ experiment (t-SIM-ddMS2). The exact mass of the molecular ions and their corresponding fragmentation spectra were compared with the entries in the lipidomic database LIPIDMAPS. For each group of TAG molecular species, the chemical formula of the neutral mass was calculated. Their classification was based on the number of carbons of the fatty acid residues (CN, TG x:−) and the number of double bonds in the fatty acid residues (DB, TG −:y), as shown in Table 2. The 14 groups of TAG molecular species were identified after fragmentation and determination of all FA moieties present in each group. The fragmentation spectra were compared with the theoretical spectra generated in LIPIDMAPS to characterize the groups of target molecules.

From Table 2, it can be derived that the fatty acid moieties identified in the TAG molecular species were characterized by a high abundance of unsaturated fatty acids, mainly oleic, linoleic, and linolenic acid. They were contained in the TAG molecular species present at higher concentrations in the hay milk samples. This was confirmed by the findings of the work of Bugaud et al., in which hay milk contained higher quantities of polyunsaturated FA [32]. Indeed, milk obtained from cows fed with diets rich in hay have an increased content of linolenic acid [33,34], whereas diets including maize silage lead to milk richer in short-chain FA, as well as myristic, palmitic, stearic, and oleic acid [35]. Diets rich in grass silage increase the content of myristic and palmitic acid at the expense of mono- and polyunsaturated FA [7]. It has been reported that the concentration of α-linolenic acid in milk obtained with silages generally decreases [7].

The higher relative abundancies of unsaturated fatty acid residues in the target TAGs reflects the cows’ diet. In silage-based diets, their decrease could also correlate with the fermentative activity of LAB [36], but other factors influence the final FA composition of milk fat, and LAB activity could be only one of those. In order to consider all those factors, a group of markers, like the 14 groups of target TAGs, represents a promising approach.

### 3.4. Discrimination of Milk Samples Using Multivariate Statistical Analysis

We assessed the 14 groups of target TAGs for the discrimination of the type of milk (hay milk (HM), milk from silage (SM-G/M)). The 14 groups of target TAG molecular species were acquired in targeted single ion monitoring (SIM) mode. For each TAG group in all milk samples, the resulting peaks were integrated from an extracted ion chromatogram (XIC). The relative intensities were used for statistical analysis and discrimination of the samples. 

**Figure 4:**
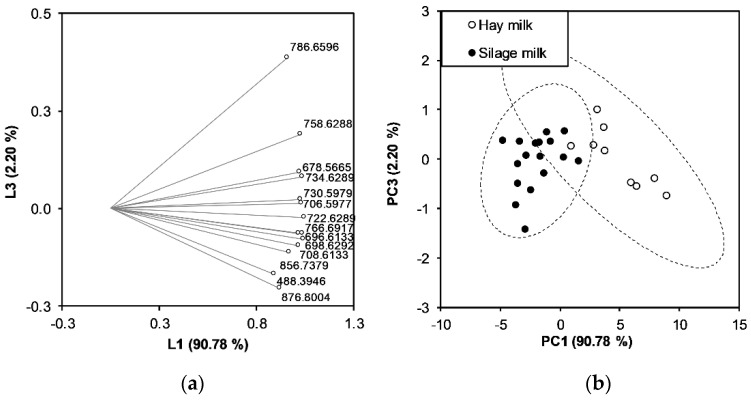
Principal component analysis of target TAGs obtained from the analysis of the hay milk and silage milk (milk obtained from cows fed with grass or maize silage in the ration) samples. Of the total variance, 92.98% is explained by the first and the third principal component. Loading of the 14 variables representing the target TAGs (**a**). Score plot showing the samples separated according to the type of milk produced (hay milk, silage milk) (**b**).

Principal component analysis (PCA) was first performed using the areas of the target TAG groups. We evaluated whether the selected variables could be fitted to build discrimination models. From the PCA, the first and the third principal component explained 92.98% of the total variance and could display the data structure (Figure 4). The loading plot (Figure 4a) shows the relationship between the variables and how much they influence the system. It was possible to observe that the 14 target groups of TAGs form a group based on which the score plot can be built. The score plot shows a separation of the samples into distinct groups according to the type of milk for the samples considered in this experimental plan (Figure 4b: hay milk and milk from silage). The hay milk samples are located on the positive side of the PC1, which indicates higher amounts of the selected TAGs in these samples.

Next, the capacity of the target TAGs to predict the type of milk was assessed using the linear discriminant analysis (LDA) model based on two classes representing the type of milk: hay milk and milk from silage. The sample set was divided into a training sample and a validation sample, with final cross validation using the leave one out (LOO) algorithm. The LDA gave an overall recognition percentage of 100% (error rate 0%, the same for the LOO cross-validation). All milk samples were classified correctly according to their type based on silage and hay feeding during the winter-feeding period considered in our experimental plan (Table 3). 

**Figure 5:**
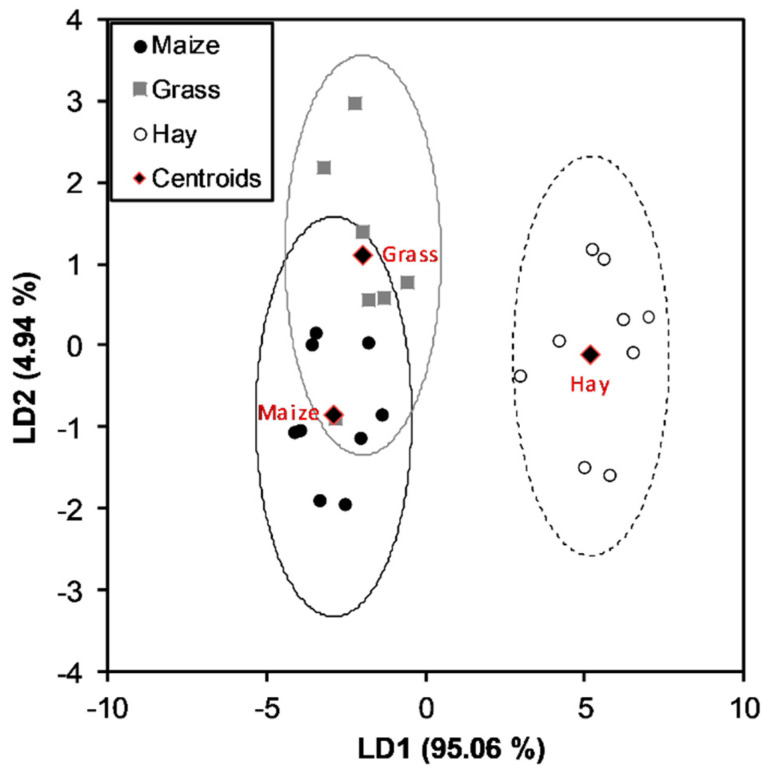
Linear discriminant analysis of the marker TAGs integrated areas of the milk samples according to the implemented feed in the ration (hay, grass silage, and maize silage).

Then, we used the prediction model to determine whether the model could also predict the type of silage implemented in the ration for bovine feeding (grass or maize silage). Therefore, the three types of feed in the ration (hay, grass silage, and maize silage) were selected as classes. LDA showed that the first two canonical functions could classify the observations between groups. Figure 5 shows the corresponding canonical score plot in which the samples were grouped according to the class, i.e., hay milk, grass silage, and maize silage. 

The LDA classification model was also repeated, considering the silage sub-classes maize silage and grass silage (Table 3). The model had an overall recognition of 92% for the fitting and LOO cross-validation.

### 3.5. Comparison between DHSA and TAGs 

Finally, the results of the GC-MS method and the HPLC-HRMS method were compared regarding their ability to discriminate the milk samples based on the type of feed. For the GC-MS method, the identity of the milk samples was assessed by the presence of DHSA. When this CPFA was present in the sample, it could directly be linked to the presence of silage in the ration. However, this was only the case with the SM-M samples, whereas not all SM-G samples were affirmative for DHSA (Table 1). As shown in Table 3, all HM samples were classified as such; all SM-M samples but not all SM-G samples were classified correctly with the GC-MS method. The absence of DHSA could therefore not be used as an indicator of hay milk when SM-G samples were also considered. When constructing a classification model using the presence of DHSA as an indicator of silage in the ration, in overall 84% of the milk samples were assigned correctly. In comparison, a higher recognition percentage was obtained with the LDA classification models using the HPLC-HRMS method. This was demonstrated by the 100% recognition obtained with the classification model based on the target TAGs (Table 3).

Overall, the HPLC-HRMS method resulted in better discrimination of the type of feed than the GC-MS method for the winter feeding period considered in the experimental plan. The untargeted approach benefited from the high resolution of HRMS, which could provide a detailed profile of each milk sample [37]. The variability between the profiles was better caught thanks to the non-targeted approach combined with multivariate analysis. Furthermore, building a prediction model based on a group of markers, rather than a single marker, was less susceptible to variations derived from the heterogeneity of the sample set.

## 4. Conclusions

This study proposed an HPLC-HRMS approach for the detection and identification of markers or groups of markers to assess the authenticity of hay milk in comparison to the targeted GC-MS method. This investigation included 27 samples collected during one winter season. HPLC-HRMS resulted in the identification of 14 groups of target TAG molecular species able to discriminate the type of implemented feed in the ration for milk production. Classification models based on LDA could predict the presence of silage in the ration with 100% recognition. Good comparability of the HPLC-HRMS method with the target GC-MS method using DHSA as marker was obtained when considering the HM samples vs. the SM-M samples. However, when also considering SM-G samples, a better recognition percentage was obtained with the target TAGs than with DHSA. The target TAGs might not account for the eventual presence or absence of DHSA, but on other dietary factors affecting the FA profile of the milk. Ultimately, by using a group of TAG markers, rather than a single marker, and with the aid of multivariate analysis, the variability in the milk sample set could be correlated to the presence of any silage (maize or grass) in the ration. To confirm the validity of the method, a bigger data set will be needed including samples from summer and winter seasons from at least two years.

## Figures and Tables

**Table 1:** Quantification of dihydrosterculic acid (DHSA) (average ± standard deviation, *n* = 3) in milk samples using GC-MS.

Milk Sample	Farm	DHSA (mg/kg Fat)	RSD (%)
HM	A	<LOD	
	B	<LOD	
	C	<LOD	
SM-M	D	94 ± 13	13
	E	94 ± 68	72
	F	52 ± 10	19
SM-G	G	<LOQ	
	H	<LOD	
	I	30 ± 11	28

HM = hay milk, SM-G = milk obtained from cows fed with grass silage in the ration, SM-M = milk obtained from cows fed with maize silage in the ration. LOD (Limit of detection) = 7.5 mg/kg fat, LOQ (Limit of quantification) = 25.0 mg/kg fat, RSD, relative standard deviation.

**Table 2:** Classification of the 14 groups of target TAG molecular species and tentative identification of their FA moieties.

*m*/*z* [M+NH_4_]^+^	Predicted Formula	Classif. (CN:DB *)	*m*/*z* Fragments	Tentatively Identified Fatty Acid Moieties
488.3946	C_27_H_50_O_6_	TG 24:0	355.2843, 327.253, 299.2217, 155.143, 127.1117, 109.1012, 99.0804, 81.0699	butyric (4:0); caproic (6:0); caprylic (8:0)
678.5665	C_41_H_73_O_6_	TG 38:3	573.4869, 405.3011, 383.3157, 261.2213, 239.2355, 71.04924	butyric (4:0); palmitic (16:0); linolenic (18:3)
696.6133	C_42_H_78_O_6_	TG 39:1	591.5349, 437.3627, 409.3312, 409.3258, 397.3312, 397.3220, 265.2524, 99.0804, 71.0492	butyric (4:0); caproic (6:0); pentadecanoic (15:0); margaric (17:0); oleic (18:1)
698.6292	C_42_H_80_O_6_	TG 39:0	593.5503, 565.5195, 509.4567, 453.3939, 439.3783, 425.3625, 411.3469, 397.3313, 313.2728, 267.2680, 253.2525, 239.2368, 235.2419, 225.2211, 221.2262, 211.2056, 207.2107, 193.1951, 173.1171, 155.1431, 137.1325, 127.1117, 99.0804, 81.0699, 71.0855, 53.0025	butyric (4:0); caproic (6:0); caprylic (8:0), capric (10:0); pentadecanoic (15:0); margaric (17:0); stearic (18:0)
706.5977	C_43_H_76_O_6_	TG 40:3	409.3316,407.3159, 99.0805, 411.3470, 601.5199, 265.2528, 145.0860, 263.2371, 261.2213, 405.3002, 433.3314, 119.0857, 127.1118, 573.4892, 247.2422, 245.2266, 243.2115, 239.2368, 173.1324, 313.2731, 339.2894, 155.1433, 53.0026, 99.1169, 71.0492	butyric (4:0); caproic (6:0); palmitic (16:0); stearic (18:0); oleic (18:1); linoleic (18:2); linolenic (18:3)
708.6133	C_43_H_78_O_6_	TG 40:2	603.5757, 409.3601, 339.2887, 265.2522, 247.2463, 145.1018, 71.04978	butyric (4:0); oleic (18:1)
722.6289	C_44_H_80_O_6_	TG 41:2	589.5209, 435.3455, 425.3624	caproic (6:0); margaric (17:0); linoleic (18:2);
730.5979	C_45_H_76_O_6_	TG42:5	625.5178, 457.3311, 383.3153, 313.2517, 239.2368, 145.1012, 71.0492	docosapentaenoic (22:5); palmitic (16:0); butyric (4:0)
734.6289	C_45_H_80_O_6_	TG 42:3	717.6027, 601.5206, 435.3476, 437.3621, 265.2532, 263.2367, 99.0805, 81.0699	caproic (6:0); oleic (18:1); linoleic (18:2)
758.6288	C_47_H_80_O_6_	TG 44:5	411.3466, 285.0094, 239.0951, 201.1638, 109.1014	caprylic (8:0); capric (10:0); lauric (12:0); myristic (14:0); docosapentaenoic (22:5);
766.6917	C_47_H_88_O_6_	TG 44:1	605.5507, 577.5189, 549.4877, 523.4722, 521.4564, 493.4251, 467.4093, 465.3938, 109.1011, 95.0854, 85.1011 81.0698, 71.0855, 57.0701	caprylic (8:0); capric (10:0); lauric (12:0); myristoleic (14:1); myristic (14:0); palmitic (16:0); oleic (18:1); stearic (18:0)
786.6596	C_49_H_84_O_6_	TG 46:5	569.456, 313.278256, 467.4245, 239.2007, 211.2057	linoleic (12:0); myristic (14:0); docosapentaenoic (22:5); eicosapentaenoic (20:5)
856.7379	C_54_H_94_O_6_	TG 51:5	639.5344, 571.4151, 537.4889, 507.1079, 373,7151 239.2007, 299.2578, 191.1788	lauric (12:0); myristoleic (14:1); pentadecenoic (15:1); myristic (17:0); myristoleic (17:1); docosapentaenoic (22:5); docosatetraenoic (22:4); docosatrienoic (22:3)
876.8004	C_55_H_102_O_6_	TG 52:2	221.2262, 239.2367, 245.2261, 263.2366, 267.2684, 313.2734, 575.5031, 579.5311, 603.5344	linoleic (18:2); stearic (18:0); palmitic (16:0)

* CN:DB = carbon number: total double bond number, of the 3 FA.

**Table 3:** Prediction of the type of feed used in the rations of cows based on target TAGs with LDA classification model and based on the presence of DHSA applied in milk. Rows represent the true class; columns represent the assigned class. Percentages of correct classified samples appear in brackets.

	Class	Hay	Silage	Sub-Class	Grass	Maize	Total
Fitting	Hay	9 (100%)	0 (%)		0 (%)	0 (%)	9
	Silage	0 (%)	18 (100%)	Grass	8 (89%)	1 (11%)	18
				Maize	1 (11%)	8 (89%)	
Cross validation, leave one out	Hay	9 (100%)	0 (%)		0 (%)	0 (%)	9
	Silage	0 (%)	18 (100%)	Grass	8 (89%)	1 (11%)	18
				Maize	1 (11%)	8 (89%)	
DHSA present	Hay	9 (100%)	0 (%)		0 (%)	0 (%)	9
	Silage	4 (22%)	14 (78%)	Grass	5 (56%)	-	18
				Maize	-	9 (100%)	

## Data Availability

The data presented in this study are available on request from the corresponding author.

## Supplementary Materials

The following are available online at https://www.mdpi.com/article/10.3390/foods10122926/s1, Table S1: title, Mass list to match 253 groups of TAG molecular species with the same number of carbons (CN) and number of double bonds (DB) in the FA residues with the entries of the peak table generated in Compound Discoverer.
